# A Spatial Transcriptomics Browser for Discovering Gene Expression Landscapes across Microscopic Tissue Sections

**DOI:** 10.3390/cimb46050284

**Published:** 2024-05-13

**Authors:** Maria Schmidt, Susanna Avagyan, Kristin Reiche, Hans Binder, Henry Loeffler-Wirth

**Affiliations:** 1 Interdisciplinary Centre for Bioinformatics (IZBI), Leipzig University, Härtelstr. 16-18, 04107 Leipzig, Germany; schmidt@izbi.uni-leipzig.de (M.S.); binder@izbi.uni-leipzig.de (H.B.); 2 Armenian Bioinformatics Institute, 3/6 Nelson Stepanyan Str., Yerevan 0062, Armenia; 3 Department of Diagnostics, Fraunhofer Institute for Cell Therapy and Immunology (IZI), Perlickstrasse 1, 04103 Leipzig, Germany; 4 Institute for Clinical Immunology, University Hospital of Leipzig, 04103 Leipzig, Germany

**Keywords:** molecular biology, melanoma, mouse brain, spatial gene set analysis, receptor–ligand interactions, self-organizing map (SOM) machine learning, intra-tumoral heterogeneity microanatomy, 10x Visium technology

## Abstract

A crucial feature of life is its spatial organization and compartmentalization on the molecular, cellular, and tissue levels. Spatial transcriptomics (ST) technology has opened a new chapter of the sequencing revolution, emerging rapidly with transformative effects across biology. This technique produces extensive and complex sequencing data, raising the need for computational methods for their comprehensive analysis and interpretation. We developed the ST browser web tool for the interactive discovery of ST images, focusing on different functional aspects such as single gene expression, the expression of functional gene sets, as well as the inspection of the spatial patterns of cell–cell interactions. As a unique feature, our tool applies self-organizing map (SOM) machine learning to the ST data. Our SOM data portrayal method generates individual gene expression landscapes for each spot in the ST image, enabling its downstream analysis with high resolution. The performance of the spatial browser is demonstrated by disentangling the intra-tumoral heterogeneity of melanoma and the microarchitecture of the mouse brain. The integration of machine-learning-based SOM portrayal into an interactive ST analysis environment opens novel perspectives for the comprehensive knowledge mining of the organization and interactions of cellular ecosystems.

## 1. Introduction

Molecular diagnostics of transcriptional activity in tissue biopsies has experienced multiple technical revolutions in the last two decades. Initially, surface hybridization-based microarrays allowed for targeted profiling of more than 20,000 human genes per chip at moderate costs [1]. These were later superseded by high-throughput DNA and RNA sequencing [2], which facilitated the expression profiling and also untargeted detection of novel transcripts, depending on sequencing depth [3,4]. Both techniques require a significant amount of sample material for extraction of the desired RNA, which is provided by bulk samples, usually containing pooled populations of hundreds to millions of individual cells. This entails the problem of intermixing expression signatures, as cells from different tissues, different cell types, and potentially diseased and healthy cells are captured together by the biopsy. A further problem is the sampling bias caused by intra-tumor heterogeneity [5,6]. Emerging single-cell isolation methods overcame these issues and delivered the individual cells’ transcriptomes [7]. Hence, transcriptomics is a child of technological progress, from the microarrays that first enabled genome-scale experiments to high-throughput sequencing and, afterward, the revolution triggered by single-cell methods.

However, these dissociation-based techniques share a major drawback: the loss of spatial information, crucial for understanding tissue functionality. Recent developments in sequencing technologies overcame this gap by resolving the spatial information of transcriptomes on a microscopic scale [8]. For example, the Visium spatial transcriptomics (ST) technique uses sequencing and localization barcoding [9] to analyze elementary spatial units of about 55 μm in diameter, called ‘spots’. Each spot contains a few (up to about a dozen) cells enabling a sort of ST microscopy (Greek ‘mikros’—small—and ‘skopeo’—look at [10]).

The field of ST is now developing rapidly, with a potentially transformative effect across many areas of biology [11]. Spatial resolution will be vital for scientific questions such as understanding the complex ecosystem of the tumor microenvironment (TME), or the cellular architecture of organismal development and the resulting microanatomy of complex healthy tissues such as the brain. The wet lab technologies that produce big (i.e., very large and complex) sequencing data urgently require computational methods for their comprehensive analysis and interpretation. Single-cell sequencing previously led to an explosion of computational tools, ranging from adaptations of bulk omics methods to inventions of novel machine learning approaches for pseudotime and RNA velocity analyses. Now, the spatial field is similarly poised for a period of rapid and exciting progress in bioinformatics and systems biology [12].

So far, the development of computational methods for spatial omics has focused on preprocessing, read mapping, and quality control tasks, facilitated by tools such as Space Ranger [13], ST Pipeline [14], or SnapATAC [15]. Next-step tools like Seurat [16], BayesSpace [17], and SpaGCN [18] accomplish class discovery tasks, providing insights into cell types and cellular subpopulations. Other questions, such as spatially-variable gene identification, benefit from tools like SpatialDE [19], trendsceek [20], and SPARK [21], with each offering unique strategies for pinpointing genes with distinct spatial expression patterns. Further tools like CARD [22], Tangram [23], SpaGE [24], GCNG [25], SpaOTsc [26], MULTILAYER [27], stLearn [28], SpaRx [29], SiGra [30], and SpatialData [31] address diverse issues ranging from cell-type deconvolution in the capture spots to the exploration of cell–cell interactions, and also support region annotation, drug response, and spatial trajectory analyses. These tools collectively provide a first (by far not complete) set of bioinformatics tools enhancing our understanding of spatial transcriptomics data. Recent reviews provide a wide overview of the state of the art of the field (see [8,32,33,34] and references cited therein).

Traditional optical microscopy requires ‘skopus’ functionalities to ‘look at’ the specimen, i.e., its detailed visual inspection, usually through its eyepiece, enabling active manipulations such as shifting and zooming the inspected region of the microscopic slide. This process often incorporates a variety of histochemical staining, e.g., for pathological inspections. In ST, such ‘skopus’ tasks are accomplished computationally and have the need for versatile platforms, advanced data exploration capabilities, user-friendly interfaces, and knowledge mining of spatial information, such as that provided partly by the ‘Loupe Browser’ [35] for 10x Genomics data.

We here present a newly developed ST web tool, designed for the interactive discovery of ST images under various functional aspects. A central novel feature that makes our tool unique is the application of self-organizing map (SOM) machine learning to ST data, which generates individual expression portraits for each of the spots in the ST image, enabling combined ‘skopus’ tasks in the transcriptome landscape and the ST image as well. These portraits provide detailed information about the local expression landscape with individual spot resolution in an easily perceivable and interpretable fashion. Our browser enables users to investigate single gene expression by selecting any of the more than ten thousand genes in the dataset, explore the expression of functional gene signatures by selecting them from a repository of a few thousand gene sets implemented in the tool, as well as inspect the joint expression of receptor–ligand pairs to study the spatial patterns of cell–cell interactions.

The method of data portrayal through SOMs has been previously developed for dimension and redundancy reduction in multidimensional omics data [36], making use of the algorithm of Kohonen maps [37]. It was previously applied by us to a wide spectrum of bulk omics and single-cell data using genetic, transcriptomic, and epigenetic data, and to their integrative multi-omics analysis (see, e.g., [38,39,40,41,42]). The method has been proven effective for the modularization and functional interpretation of cellular programs and, as its specific feature, enables an easily perceivable and interpretable visualization of granular data landscapes [43]. Our ST application makes use of the previously developed ‘oposSOM’ software (version 2.4) [44]. The ST browser is offered as a novel extension of the oposSOM-Browser [45] developed for bulk and single-cell omics data. In this publication, we describe the functionality of the spatial browser in the context of two use cases, addressing in detail the intra-tumoral heterogeneity of melanoma and, as an illustration, the cellular architecture of the mouse brain. We focus on the application aspects and on biological knowledge mining at the gene and cell levels to illustrate the association between spatial and functional aspects for the two selected use cases.

## 2. Materials and Methods

### 2.1. The Spatial oposSOM-Browser: Overview and Availability

**Figure 1:**
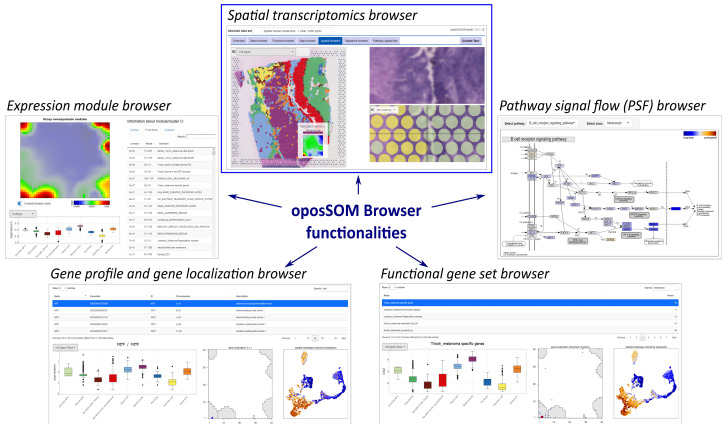
The spatial transcriptomics browser is a novel functionality of the interactive oposSOM-Browser data mining tool [45], providing various options for gene profiling and function mining of ST data such as coloring of the images according to expression levels of selected genes, gene signatures, receptor–ligand interactions, and clusters of spots. It supplements already implemented modules of the oposSOM-Browser such as the expression module browser (providing details of the SOM expression landscape), pathway signal flow (PSF) browser (providing class-specific activation topologies of KEGG pathways), as well as gene and functional gene set browsers.

The spatial transcriptomics (ST) browser is a novel bioinformatics tool enabling the interactive analysis of microscopic tissue slices using transcriptomic data. The tool is embedded as an ST module in the oposSOM-Browser, which has been previously developed for the interactive exploration of omics data processed by the SOM portrayal method ([45], Figure 1). The ST browser adds functions that enable the detailed interactive analysis of data obtained by the spatial mRNA capture technology of microscopic tissue sections, resolving them into spots of sizes of about 55 × 55 μm containing typically up to one dozen single cells. Adaptations to single-cell resolved ST technologies such as Visium HD are presently under way.

As a unique feature, the ST browser provides an individual SOM image of each spot, which serves as a local fingerprint of its transcriptomic landscape [44]. For a detailed description of the SOM portrayal method and its applications, we refer to our previous publications (method: [36,46]; selected applications: [41,43,47]). The SOM portrayal of ST data resembles previous SOM portrayals of bulk transcriptomics samples, where each spatial spot represents a ‘micro-bulk’ sample capturing the transcriptomes of 7–15 cells. For interactive SOM space discovery, one can use the previously described functions of the oposSOM-Browser [45], while the discovery of the transcriptome in spatial coordinates is provided by the novel ST browser module.

The spatial browser is implemented as an R-Shiny application [48] and can be accessed via standard web browsers under www.izbi.uni-leipzig.de/opossom-browser (accessed on 2 May 2024). Currently, the ST browser provides the two use cases described below and four additional ST samples of cancer and healthy tissues. The input data are split into different data types which require several preprocessing steps (see next subsection). To avoid performance problems, usually related to the SOM processing of ST data, we offer the implementation of external data via support by the authors. Interested users are invited to provide their analyses to the browser via request to the corresponding author. The workflow together with a list of the required data for integration into the oposSOM-Browser is given as Supplementary Figure S1. Note that input data for the ST browser can be used for independent downstream analyses by applying additional methods such pseudotime and RNA velocity analyses for identifying developmental trajectories in the ST images.

### 2.2. Input Data, Preprocessing, and SOM Portrayal of the Spatial Transcriptome

Input data for the browser comprise the ST image, the preprocessed spot data (e.g., as Seurat R-object [49]), and the SOM-processed spot-level data as an R-object using the package ‘oposSOM’ available on Bioconductor and Github [44] (see Figure S1 for an overview of input data requirements). Processing of an ST use case with a few thousand spots requires about 12–48 h of runtime on a standard scientific laptop (Intel i7 CPU, 16 GB memory), covering SOM training and subsequent statistical routines that are implemented in oposSOM.

We apply self-organizing map (SOM) machine learning to the normalized and centralized ST data expression data in logarithmic scale, where each of the spots is considered as a single ‘micro-bulk’ transcriptome sample. The expression values of each gene across all spots are considered as its expression profile. SOM processing clusters these expression profiles into 50 × 50 = 2500 so-called metagene profiles and distributes them in a two-dimensional quadratic grid using Euclidian distance similarity metrics [36]. The transcriptomic patterns of each spot are visualized as ‘expression portraits’ by color-coding the metagene expression values in the square grid topology using a red-to-blue color scale for coding over- and underexpression, respectively. Importantly, all expression portraits of the different spots can be directly compared as the localization of the genes in the SOM grid is fixed at the same position in all portraits. Mean portraits, e.g., per cell type or spot cluster, were obtained by averaging the metagene expression values across all members of a given group of spots in the respective image.

### 2.3. Downstream Analysis and Function Mining of the Spatial Images and SOM Portraits

Downstream analysis functions are implemented in the oposSOM-Browser, comprising gene and biological function analyses with spatial resolution as well as pathway mining on the class level (Figure 1 and [45]). The ST browser also provides detailed spot-level information when hovering over the image for zoomed-in views on every spot-related SOM portrait (Figures S2 and S3).

**Function mining of the spatial images** is performed by coloring them using several options, namely by selecting the expression levels of (i) single genes, (ii) gene sets, (iii) gene modules identified in SOM analysis (i.e., sets of genes extracted from the modules), and (iv) receptor–ligand interactions in terms of co-expression of the receptor and ligand pairs taken from a predefined list of genes referring to distinct pathways. Options (i)–(iii) color code the spatial spots in brown to blue for high to low expression, respectively (see below), and for option (iv), we use triple coloring with apricot/blue/green for the co-expression of the receptor and ligand or the expression of only the receptor or only the ligand, respectively. Color saturation then scales with the expression level. Receptor–ligand interaction (RLI) pairs can be selected either individually or related to pathways considering respective sets. RLs were obtained from ‘omnipath database’ [50].

**Function mining of the SOM expression portraits** is performed by visual inspection of different types of maps. The SOM algorithm self-organizes co-expressed metagene profiles into red ‘spot’-like activation patterns in the portraits. The genes included in these expression modules were extracted from the SOM portraits as gene lists as described previously [36]. Gene set enrichment and overexpression analysis based on a large collection of functional gene signatures implemented in oposSOM [44] then provides functional information about the genes in each of the modules. Another complementary option for functional mining is the mapping of the genes of a gene set into the SOM, resulting in so-called gene set maps for direct comparison with the expression patterns observed in the portraits. The pathway signal flow (PSF) algorithm provides a third option for functional analysis, tracking transcriptional activity through more than 50 KEGG pathways under consideration of the pathway topology [51].

### 2.4. Use Case Datasets: Human Melanoma and Mouse Brain

We implemented two ST datasets as use cases to illustrate the functions of the spatial browser: (i) A microscopic section of human (malignant skin) melanoma with 18,051 genes in 3458 spots as an example of the microarchitecture of cancer tissue expressing high molecular and cellular heterogeneity. (ii) The second use case dataset was chosen to describe the microanatomy of a healthy mouse brain as an example of a microscopically well-structured tissue. It consists of the profiles of 14,858 genes in 2696 spots. Both datasets were generated by 10x technology and are publicly available on the 10x Genomics website [52]. The datasets were preprocessed using the Seurat [16,49] and oposSOM [44] packages. Seurat provides the standard UMAP projection of the spots, cell type assignment, and spot clusters using the default Louvain algorithm. Further datasets are presently in preparation and will be released together with the accompanying scientific publications.

## 3. Results

### 3.1. Browsing the Spatially Resolved SOM Portraits of Melanoma

**Figure 2:**
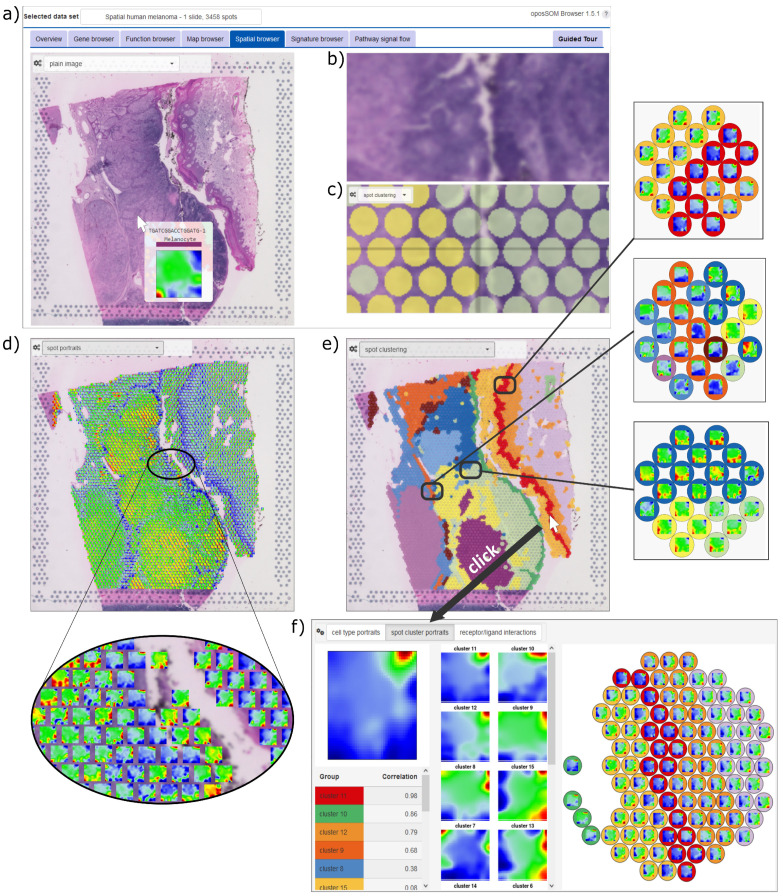
Data portrayal applies SOM to ST spots: (**a**) Screenshot of the H&E-stained (plain) image of the melanoma sample in the oposSOM-Browser. Hovering over the image with the cursor displays the expression portrait of the selected ST spot at the cursor’s position. (**b**) An enlarged view of the H&E image and (**c**) a second zoomed panel shows the cell type/cluster assignments of the spots as colored circles. (**d**) The ST image shows the SOM portraits of each of the spots as indicated in the enlargement. (**e**) Segmentation of the ST image into Seurat clusters enables zoomed-in discovery of the spot portrait environment at the cursor position. Three examples are shown on the right. (**f**) Clicking on a spot in the image opens a window that shows the spot portrait, the cell cluster archetypic portraits, a correlation-ranked list of cell types, and a zoomed-in image showing the portraits of the neighboring spots.

Spatial transcriptomics functionalities of the ‘oposSOM-Browser’ are illustrated for the melanoma use case in Figure 2a–d. Hovering over the image shows the SOM expression portrait of the spot at cursors position together with an enlargement enabling to compare the plain and the spot images (Figure 2a–c). An overview of the spot portraits is available by selecting the ‘spot portrait’ option of image visualization (Figure 2d). The image can alternatively be colored by choosing ‘spot clusters’ or ‘cell types’. The former option is usually taken from standard clustering as provided by preprocessing using Seurat, while the latter one typically results from transfer learning based on cell-type-specific marker genes. Spot clustering, for example, segments the ST image into areas of different functional impact, which identifies fine structures referring to the dominant cell type present in the spots (Figure 2e, see next subsection). Clicking on a spot opens a zoomed-in view at the cursor position showing the local portraits of the selected spot and of its neighbors, as well as their cluster memberships as colored circles (Figure 2f). Notably, the individual spot portraits slightly differ due to variations in the cell compositions and/or transcriptional programs in the respective spots, even if assigned to the same cluster. Their inspection thus provides an overview of the local heterogeneity of activated transcriptional programs with single-spot resolution. Contiguous spots, especially if they belong to different clusters, can express SOM portraits sharing common transcriptomic patterns due to the changing and partly overlapping cell states (see, e.g., Figure 2e,f). Hence, the portrayal with ‘individual’ single-spot resolution in combination with the functional interpretation of the portraits (see below) enables the spot-by-spot discovery of the image, particularly in areas of changing compositions.

### 3.2. Spot Clusters and SOM Portrayal Stratify the ST Images into Major Transcriptional Types

Clustering is a common and very useful concept to stratify high-dimensional data and, particularly, to segment the spots of ST images into areas of different cell compositions and/or transcriptional states of the cells. There is usually no gold standard for the right choice of clusters. Instead, clustering depends on the biological question pursued in a study. For ST data, it usually requires a combined evaluation including (i) statistical and bioinformatics criteria such as silhouette plots (available in the standard oposSOM-reports) or consensus clustering approaches, (ii) pathologists’ evaluation of the images, (iii) marker genes for different cell types and/or developmental stages in the case of cancers, and (iv) functional interpretation, e.g., by means of association of gene signatures with the clusters. In general, justification of the clusters chosen here for the illustration of the browser functions is beyond the scope of this publication and was previously discussed (see, e.g., [53]). In agreement with the state of the art used in numerous single-cell and ST data analyses, we applied unsupervised Louvain clustering as provided by the Seurat R-package, which stratifies the 3458 spots of the melanoma ST image into fifteen clusters labeled as c1-c15. For the association with their functional contexts and cell type composition, we used previous knowledge from numerous publications and illustrate how the browser can be used to interpret the areas and clusters identified in the ST image (see below). Moreover, hovering over the local spot portraits across the image enables the consistency of clustering to be controlled by comparing, e.g., expression patterns along the cluster boundaries (see Figure 2). Note that the clustering used in the browser is provided by the preprocessing and thus by the user’s choice.

**Figure 3:**
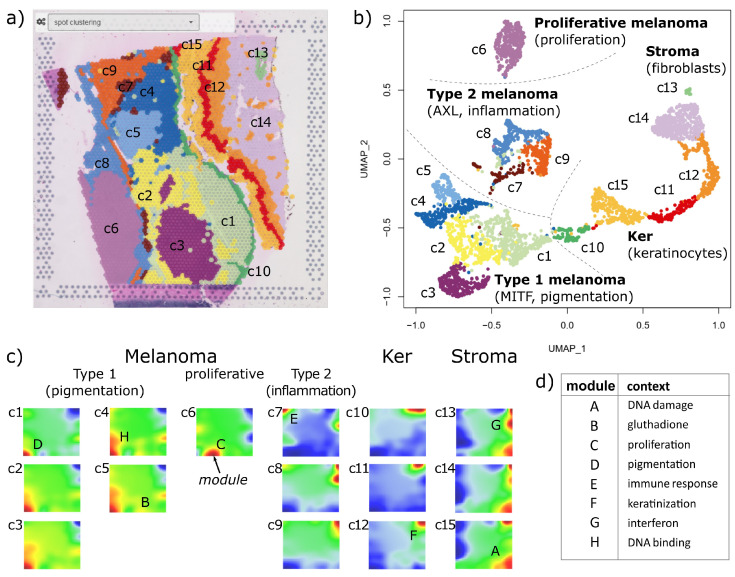
Spot cluster characteristics: (**a**) Clustering of the spots using the Louvain algorithm, as implemented in Seurat, segments the ST image into areas of different expression patterns (c1–c15). (**b**) The UMAP projection illustrates the similarity relations between the spots and the clusters. They aggregate into four major superclusters, which were assigned to type 1 (pigmentation), type 2 (inflammation), and proliferative tumor types, as well as to an epithelial cluster dominated by keratinocytes and fibroblasts. (**c**) Mean SOM portraits of the spot in each of the clusters characterize their expression landscapes. Modules of coregulated genes appear as red areas and are labeled with capital letters A–H. (**d**) Major biological context of the expression modules (see also Figure 4).

The UMAP of the spots was generated using the Seurat R-package [49]. It transforms the ST patterns into a spot-similarity plot supporting the selected clusters and, beyond this result, revealing four superclusters with different cellular and functional impacts: three of these regions relate to spots mainly containing melanoma cells with specific signatures of pigmentation, immune response, and proliferation, respectively, and one collects keratinocyte- and fibroblast-rich spots (Figure 3b). Particularly, six clusters (c1–c6) refer to different transcriptional types of melanoma cells pursuing a pigmentation (type 1) or proliferative (c6) program; three clusters (c7–c9) were assigned to inflammatory and mesenchymal melanoma cells (type 2) including their tumor microenvironmental cell communities using gene signatures taken from [54,55]; and six clusters (c10–c15) accumulate different types of bystander cells such as keratinocytes, fibroblasts, pericytes, and immune cells (T- and B-cells and macrophages) based on single-cell transcriptomics data and cell type markers taken from [56,57]. The ST image shows the spatial distribution of these clusters forming a distinct microanatomy of the tumor, where clusters of type 1, proliferative, and type 2 malignant cells form well-separated areas, respectively, which are separated from regions dominated by keratinocytes and stromal cells (Figure 3a, select the ‘spot clustering’ option in the pull-down menu). Interestingly, the UMAP spot-similarity patterns show a similar structure to the ST cluster patterns (compare Figure 3a,b), with the stroma- and keratinocyte-related regions separated from regions of type 1, type 2, and proliferative melanoma (from right to left). This result reflects the fact that the cells form intrinsically interacting communities, resulting in a similar gradient of transcriptional patterns in the ST image and UMAP projection.

SOM portrayal complements Louvain clustering of the spots by an orthogonal clustering of the gene expression profiles. In particular, each of the fifteen spot clusters is characterized by its mean expression SOM portrait obtained by averaging over all individual spot portraits of the respective cluster (Figure 3c). These portraits reveal the underlying clusters of co-regulated genes in terms of specific patterns of modules showing over- and underexpressed genes in red and blue, respectively. Overall, eight modules of co-overexpressed genes were identified and labeled with capital letters A–H. Their major functional context was evaluated using gene set analysis (Figure 3d and next subsection). Accordingly, melanoma spots of the pigmentation type uniquely express module D, which collects genes of the MITF-program active in melanocyte-like tumor cells assigned as type 1 [58]. Spots of a proliferative melanoma type express module C, which accumulates cell cycle signature genes, while inflammatory type 2 melanoma spots express module E, rich in genes of the AXL-program, along with modules originating from transcriptional signatures of the TME, such as immune T- and B-cells, macrophages, keratinocytes and fibroblasts (modules F, G and A). Keratinocyte- and fibroblast-rich spots express these latter modules in different combinations and underexpressing the melanoma-related modules C–F.

In summary, we extracted three closely related similarity patterns, namely, the ST microscopic image, the UMAP revealing similarities of the transcriptional patterns of the spots, and the SOM portraits visualizing the expression landscape of the clusters and disentangling them into modules of co-expressed genes of interpretable functional and biological context.

### 3.3. Gene Expression Modules Resolve ST Micropatterns

**Figure 4:**
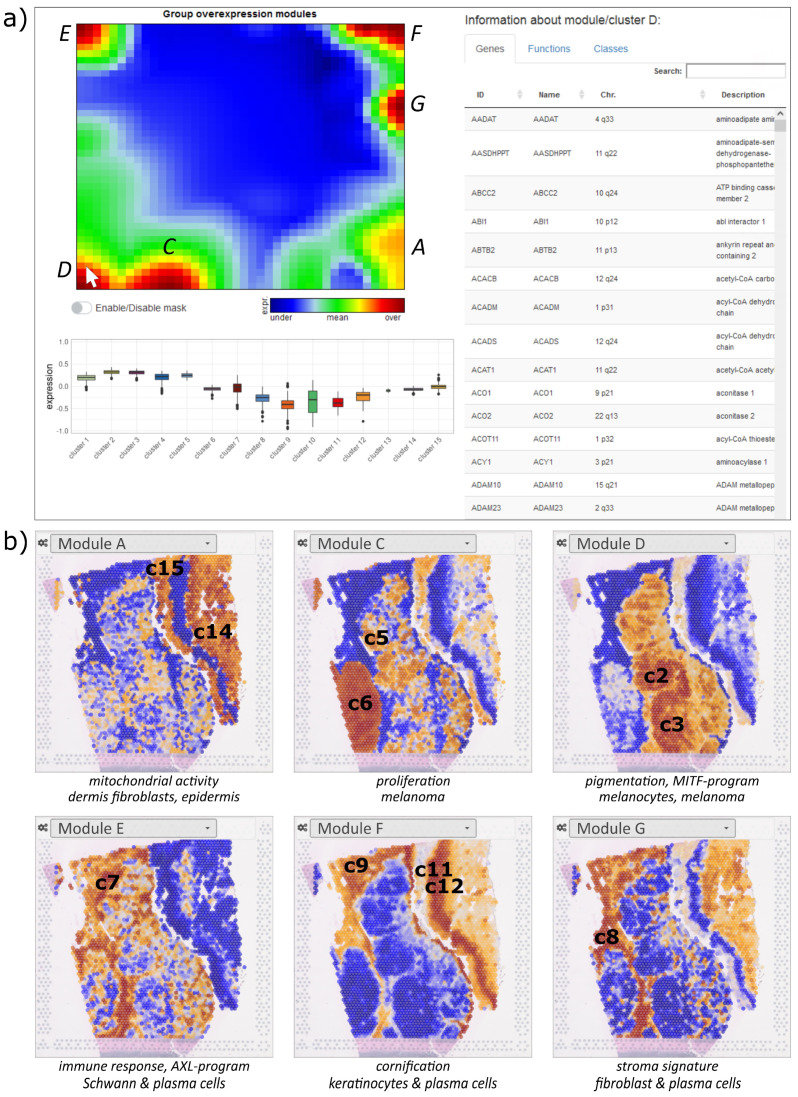
Modules of co-expressed genes and their spatial activation patterns: (**a**) The screenshot of the module browser shows the overview map (top left) and information about each module selected by clicking on the module (here module D), namely, the boxplot of its expression across the cluster (bottom left panel), and a table (right panel) listing the genes contained in the module, the enriched gene sets, as well as the activation across the cell types (in %). Modules B and H are omitted for clarity. (**b**) Module-specific spatial activation patterns reveal the underlying ST patterns. The modules relate to different cell subpopulations and biological processes, which can be further assessed in the module browser (see part (**a**)). Activated cluster numbers are indicated in the images.

SOM portrayal not only generates transcriptional landscapes for each cluster as discussed in the previous section, but also individual SOM portraits of each spot which decipher the ‘micro-bulk’ transcriptome landscapes of the up to about one dozen cells included. These portraits show characteristic module patterns that are upregulated depending on the underlying cell types and their transcriptional programs. An overview map of the observed modules is available under the ‘map browser’ section in the oposSOM menu for more detailed knowledge mining. For the spatial melanoma data, this overview map identified major modules marked with capital letters A–G (Figure 4a, module H is omitted there). Each module represents a cluster of coregulated genes, which can be inspected in the table on the right after clicking on the module of interest on the map. The table then provides the lists of genes in the module, a list of functional gene signatures enriched in the selected module, as well as the clusters activated in this module (click on the header of the column). For example, module D upregulates in melanoma cells of pigmentation type 1 and associates with the MITF transcriptional program as well as with oxidative phosphorylation (oxphos). The neighboring module C associates with DNA repair and cell cycle activity and mitochondrial activity and is assigned to proliferating melanoma cells. In contrast, module E upregulates immune response and inflammatory signatures as well as the AXL transcriptional program. Module F is activated in keratinocytes, but also in plasma cells, and associates with mucosa functions such as cornification, keratinization, and epidermis development. Module G activates in pericytes and fibroblasts and associates with collagen-containing extracellular matrix signatures, immune system processes, and epithelial–mesenchymal transition (EMT) functionality. Finally, module A associates with functions like transferase activity and DNA damage. Note that the module browser offers several module selection options such as single-spot overexpression, group overexpression (discussed here), underexpression, K-means, correlation clustering, and D-map segmentation, each considering different features of the expression landscape (see [36] for details) and, in practical consequence, enables any region in the SOM to be analyzed.

The browser enables the coloring of the ST image according to the mean expression of a selected module in each of the spots (select the module letter in the pull-down menu of the spatial browser). It visualizes spatial regions by activating or de-activating the respective module in red and blue, respectively (Figure 4b). Keratinocyte-enriched regions can be clearly distinguished from their environment (modules A, F, and G). Module C assigns highly proliferative regions of tumor cells, while module D identifies type 1 melanoma regions and module G identifies fibroblast- and endothelial-related areas. Interestingly, modules F and G both upregulate in the endothelial region but show opposite activity in the keratinocyte region on the right. It suggests a mix of keratinocytes, immune cells, and fibroblasts in the former but nearly exclusively keratinocytes in the latter region. In summary, the module browser enables a detailed functional interpretation of the expression modules, which, in turn, provides a module-based segmentation of the ST image to identify the relevant regions with information about cellular composition and association with functional knowledge.

### 3.4. Visualizing Gene and Gene Set Activities

**Figure 5:**
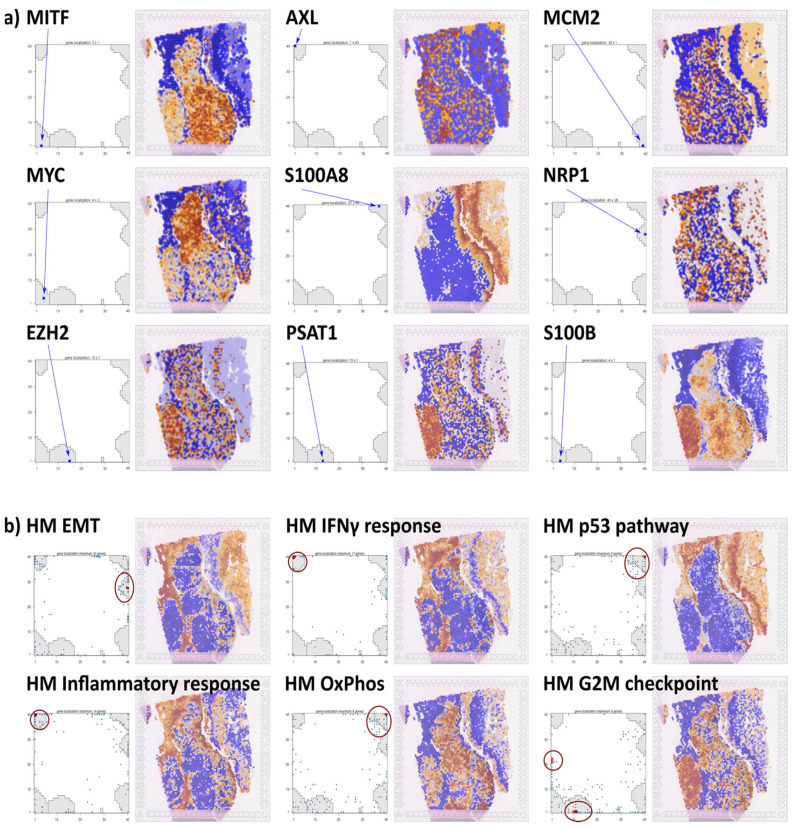
Single gene and gene set coloring of the ST image: (**a**) Selected single genes show characteristic activation patterns in the ST images. The location of each of the genes is shown separately in the gene map by blue arrows. (**b**) Coloring according to the mean expression of gene signatures taken from the category ‘hallmarks of cancer’ (HM) [65]. The gene set maps show the distribution of the signature genes in the SOM. Accumulation of genes is highlighted by red circles.

The previous section describes the coloring of the ST image according to the expression of the modules, usually including several hundreds of co-regulated genes. It visualizes the spatial patterns differing in the activation of the underlying cellular processes. In a complementary approach, one can choose the option ‘gene expression’ or ‘gene set expression’ (in the pull-down menu of the spatial browser) to visualize their expression topologies. Selected genes and gene sets are overexpressed in distinct areas of the image, e.g., AXL, MITF, and EZH2 in different melanoma cell-rich clusters (c2/c3, c4/c5, and c6, respectively), also upregulating the hallmark (HM) gene sets oxphos and G2/M checkpoint, respectively (Figure 5a,b; compare with Figure 3a). The mapping of the single gene(s) into the SOM gene map, on the other hand, reveals their location in or near modules D and C, respectively, which explains their similar ST patterns. Another group of genes and gene sets (e.g., AXL, HM angiogenesis, and HM inflammatory response) refers to the ST pattern associating with modules G and F. The gene S100A8 is reported to act as an early marker of melanoma development [59]. It upregulates in and near the keratinocyte region of the ST image (c11 and c10). Neurolipin1 (NRP1), a prognostic marker for melanoma progression [60], is activated diffusely across the type 1 melanoma areas. The gene PSAT1, together with ATF4 and NRF2, is associated with thiol starvation in melanoma [61]. Their upregulation in c6 assigns proliferative melanoma and module C to a starvation phenotype discussed earlier [62].

Other examples of knowledge mining using gene sets of immune functions and cell types, chromatin states, and previous melanoma signatures are provided in Figures S4–S6, respectively. They illustrate the intra-tumor heterogeneity of melanomas in terms of immunogenicity and epigenetic states, which, in turn, relate to the subtypes of melanomas established as inter-tumor heterogeneity in previous bulk transcriptomic studies [58,63,64]. Importantly, the ST patterns of these gene sets illustrate that bulk transcriptomics subtypes can refer to melanoma states existing in parallel in different regions of the same tumor and thus reflect not only inter- but also intra-tumoral heterogeneity (Figure S6).

These examples of knowledge mining in the ST image illustrate the analytic strength of the browser for researchers who are interested in interactively discovering the expression of single genes or functions. Selecting a module, gene set, or single gene expression enables fine-grained function browsing by applying different complementary options where the modules identify the major differential regions while the gene set and gene colorings can further specify areas in these regions due to changing cell compositions and/or cell-type-related transcriptional programs. Gene set selections allow views from different perspectives using different categories such as cancer hallmarks, GO terms for biological processes or molecular functions, and other sets taken from the literature, e.g., previous bulk and single-cell melanoma studies, immune cell signatures, and signatures of epigenetic chromatin states.

### 3.5. Spatial Distributions of Receptor–Ligand Interactions

**Figure 6:**
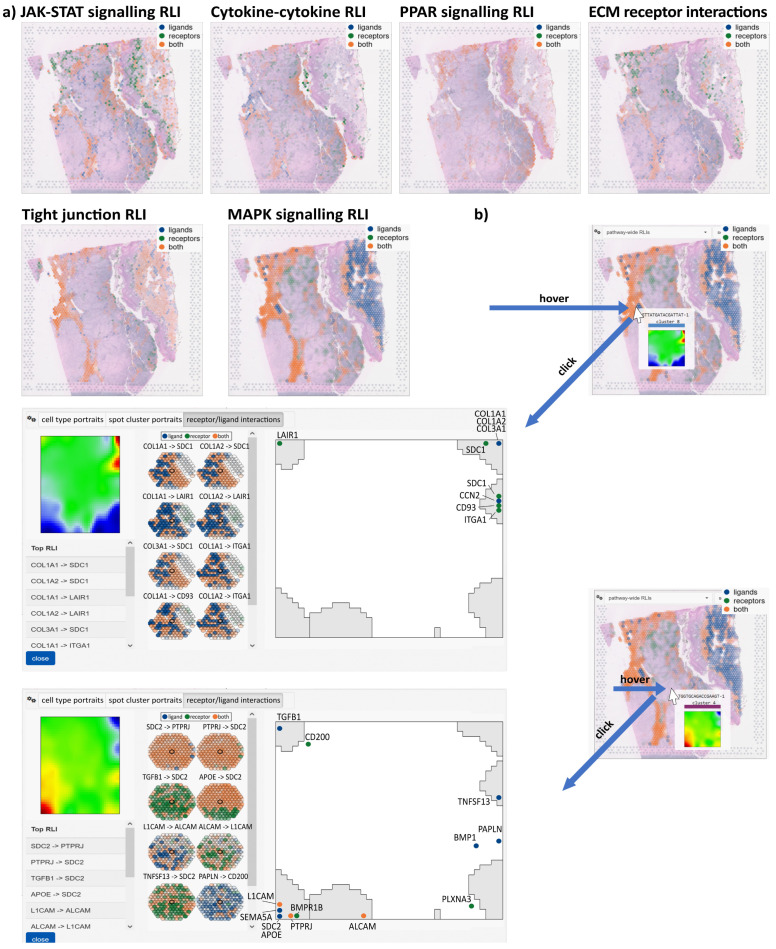
ST of receptor–ligand (R-L) interactions: (**a**) Expression mapping of R-L pairs from different pathways indicates co-expression of R and L in different areas of the image (apricot color). (**b**) Hoovering indicates the respective spot portrait. Clicking opens the R-L interactions window with the list of top expressed interactions, their spatial distribution around the selected spot, and the map of receptor and ligand genes in the SOM (from left to right).

A particular strength of ST is the ability to visualize cell–cell interactions in terms of co-expressed receptor–ligand (R-L) pairs at the transcript level. R-L pairs can be selected in the browser from predefined lists as single pairs taken from the omnipath database [50] or as a collection of pairs referring to distinct pathways downloaded from KEGG [66], Biocarta [67], and PID [68]. The browser then colors spots according to four situations, namely, if either only receptor or ligand is expressed (blue or green color, respectively), both receptor and ligand are co-expressed (apricot), or both are not expressed (no color) (Figure 6). R-L co-expression is assumed to reflect R-L-mediated cell–cell interactions in the respective spot. For example, R-Ls of different pathways (JAK-STAT, Cytokine, PPAR, ECM, and tight junctions) co-express in different areas of the map, namely, in the immune-cell-enriched and keratinocyte-enriched regions, respectively (Figure 6a, compare with Figure 3). Receptors and ligands related to the MAPK pathway co-express mainly in the immune-cell- and stroma-enriched area, while receptors alone upregulate in the fibroblast-enriched area (blue). The melanoma-enriched region shows low expression of all situation (solely receptor, solely ligand, and R-L).

To obtain a visualization of spatial transcriptomics colored by the expression of a receptor–ligand pair within a particular gene set, users should start by selecting the ‘Receptor/Ligand Interactions RLI’ option from the spatial browser’s pull-down menu. Additionally, one can select the ‘pathway-wide RLIs’ option to color the map based on the collective expression of all cell–cell interactions in a given gene set. Clicking on a selected spot in the ST image opens a window (‘receptor-ligand interactions’) which lists the top R-L-pairings, and their activity in the neighboring spots, and shows a map of the R-L genes in the SOM (Figure 6b). Most R-L genes are located in the modules upregulated in the respective SOM portraits, namely, modules F and G in the first situation and module D in the second one (Figure 6b, part above and below, respectively). This reflects the overall co-regulation of R-L genes, as expected. R-L pairs are then ranked according to joint (mean) expression. The top listed genes in keratinocyte module F include COLA1, known as a marker for poor prognosis [69], and SDC1, a gene of the syndecan family promoting the invasiveness [70] of melanomas. Another syndecan, SDC2, together with PTPRJ is on top of the R-L-list in the melanocytic module and plays a crucial role in the migratory potential of melanoma cells [71] and metastatic melanoma associated with cachexia [72], respectively. Hence, co-expression of the intended R-L gene pairs provides an analytic option for searching and visualizing cell–cell interactions in the images.

### 3.6. Cell-Type-Resolved Pathway Activities and Signature Browsing

The pathway signal flow (PSF) browser selection (Figure 1) provides directed graphs of a set of KEGG pathways, visualizing the activation patterns of the genes along the pathway branches in a spot-cluster-specific fashion. For illustration, we provide activation patterns of the MAPK and VEGF pathways in selected clusters (Figure S7 and Figure S8, respectively). MAPK is primarily activated along the TNF branch in keratinocytes, to a lesser degree in fibroblasts, and at low activity levels in type 1 melanoma spots (Figure S7). Thus, the pathway browsing function enables visualization of pathway activation with cell-type and/or cluster resolution using PSF metrics. These metrics consider directed interactions between the genes along the pathways, starting from the source towards the sink nodes [51].

### 3.7. Resolving the Microanatomy of the Mouse Brain

**Figure 7:**
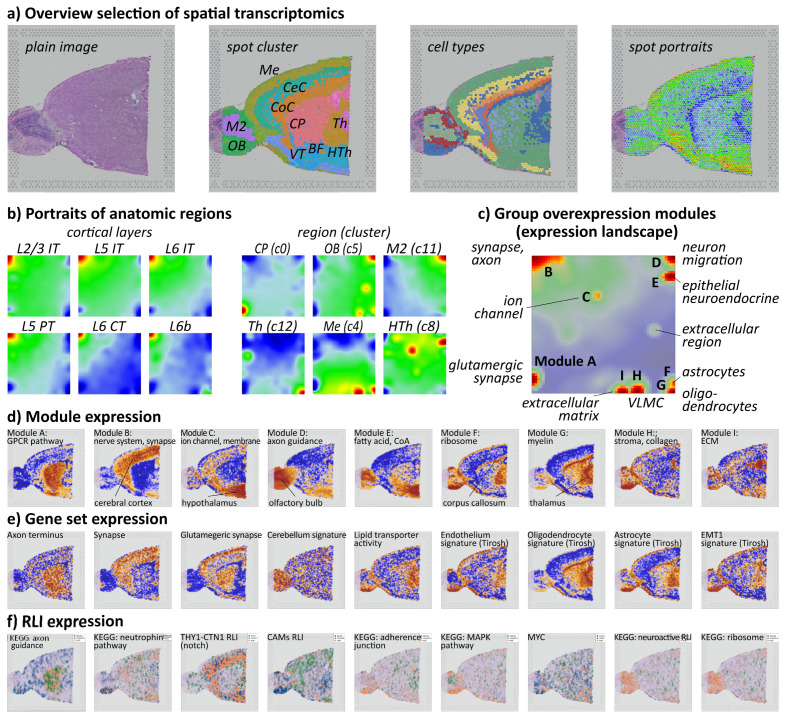
ST and SOM gene expression portrayal of the mouse brain: (**a**) Plain H&E image and cell cluster (Seurat), cell type, and spot portrait coloring of the image. (**b**) Cell-type expression portraits of neuronal and non-neuronal cells. (**c**) The module overview map provides an overview of the major expression modules labeled A–I. (**d**) Each of them transforms into a unique ST pattern. (**e**,**f**) ST of selected gene sets and receptor–ligand interactions support the functional interpretation of the microanatomy of the sample.

As a second use case of the ST browser, we selected an ST image of a mouse brain taken from [52]. The mouse brain revealed a highly complex cellular architecture essential for integrating information and interpreting the structure–function relationship at the cellular level [73]. Spot cluster and cell type coloring reveal the basic microanatomy of the brain, distinguishing regions such as the olfactory bulb (OB), the MEIS2-enriched region (M2), the meninges (Me), the cerebral cortex (CeC), the corpus callosum (CoC), the thalamus (Th), the hypothalamus (HTh), the caudate putamen (CP), the basal forebrain (BF), and the ventral stratum (VT) (Figure 7a). Region-related portraits (Figure 7b) associate with the respective ST coloring (Figure 7a). Different neurons (L2/3, L4, L5, L6) form the cortical layers with a characteristic expression of module B in the left upper corner of the SOM, which associates with synaptic- and axon-related gene expression patterns (Figure 7b,c), while neuronal cells of the thalamus express characteristic genes in module A (glutamergic synapse). Other modules associate with non-neuron cell types and structures, e.g., module C with ion channel activity; F and G with astrocytes and oligodendrocytes, respectively; and H is expressed in the meninges with collagen and vascular cells (VLMCs) [74]. Coloring of the ST images using the genes taken from the module transforms them into unique spatial patterns, which underlines their character as a sort of ‘eigen-gene’ in gene and tissue space (Figure 7d). Gene set expression and R-L coloring assign functional aspects to the spatial patterns (Figure 7e,f). More detailed information can be extracted by using the browsing functions as described in the melanoma use case and the large repertoire of implemented gene sets. Use case 2 illustrates the potential of the browser by studying spatial transcriptomics to resolve the microanatomy of healthy tissues.

## 4. Discussion

We here introduce a novel bioinformatics tool for the comprehensive, interactive study of ST data with the unique feature of applying SOM data portrayal machine learning to the spatial spot-level transcriptome data. It provides individual images of the gene expression landscape with single-spot resolution, which enables transcriptional programs to be deciphered via a bundle of browsing functions by coloring and segmenting the image based on functional aspects. The spatial architecture of the tissue under study here is the focus of the tool. In addition to the imaging of the ST data, it provides two other visualizations: a UMAP of the spots, which shows the similarity relations between the transcriptional spots (Figure 3b), and a module overview map providing an overview of the observed modules of co-regulated genes, which, in turn, decipher the cell-related activity states in each of the spots (Figure 4a). These modules enable the data-driven interpretation of the ST image without using sets of markers from external studies. This triple-clustering strategy in microscopy space, sample similarity space, and gene expression space enables the user to link directly spot clusters in the image and the UMAP with specifically up- and downregulated transcripts evident as colored patterns in the portraits.

The performance of the spatial browser is illustrated using two selected use cases from 10x Visium ST repository. The first use case demonstrates the power of the tool to explore the characteristics of complex cell communities by deciphering the intra-tumoral heterogeneity in melanoma. By employing data-driven segmentation based on expression modules, our tool extracts the relevant components, including different tumoral types, and TME- and keratinocyte-rich areas. This allows for deeper exploration through querying the expression of single genes and gene signatures within a defined functional context, as well as using co-expression information of receptor–ligand pairs. This functional segmentation of the ST image supports the clustering provided by unsupervised methods and thus, in a more general context, offers an option to verify clusters in ST images based on their biological meaning as well as the gene expression landscapes of the spatial spots. The availability of individual spot portraits enables the detailed study of subtle changes in the transcriptional landscape between adjacent spots under developmental aspects to identify and visualize possible trajectories of tumor progression in ST images. Extended analyses using pseudotime and RNA velocity methods in spatial coordinates using SOM portrayal are presently under way to better understand tumor development and/or the differentiation of healthy tissues.

Interestingly, the transcriptional signatures of melanoma extracted from previous bulk transcriptomics studies mark different regions of the ST sample. For example, marker genes for ‘low-grade melanoma’, ‘pigmentation subtype’, and ‘metastatic melanoma’ (Figure S6) all show overexpression in one and the same ST melanoma sample, but in different areas assigned to keratinocyte-rich, melanocyte-like, and proliferative melanoma by alternative signatures [54,58]. This ‘all-in-one’ result sheds an interesting light on previous melanoma subtyping. It indicates the co-existence of clones with varying cellular compositions in the TME and among the tumor cells. This diversity presumably reflects different stages of tumor development, which are associated with spatial segregation and might have an impact on treatment options [58].

The browser thus features an intuitive interface for the efficient analysis of receptor–ligand interactions with spot resolution. In the exemplary study, it allowed for a detailed examination of the key genes and pathways possibly relevant for cancer progression [62], thus allowing tumor development to be studied in time and space. This easy-to-use browser enhances our ability to understand cellular interactions in tissues, making complex spatial data more accessible for research on disease mechanisms and potential therapeutic targets.

The limitations of the present version of the browser are the relatively low number of implemented applications (six as of April 2024), the restriction to 10x Visium images, and the lack of direct upload options for interested users. Currently, we provide images addressing the microanatomy of skin structures (sebaceous and meibomian glands) and five images of colorectal cancer in addition to the use cases presented here. The tool is adjusted to 10x Visium technology and will be updated to Visium HD, enabling ST with a single-cell resolution with a spot size of 2 μm, and can prospectively be adapted to other emerging spatial transcriptomics systems. As described above, we invite interested scientists to contact the browser team to implement their own images in the browser. Options for independent upload are currently under consideration.

The second use case briefly illustrates the different options for analysis to disentangle the microanatomy of healthy tissues. We address the transcriptional diversity in the brain, which reflects a high complexity of interconnected neuronal cell types. The spatial oposSOM-Browser further hosts original ST datasets on the sebaceous gland and the meibomian gland as additional examples for discovering the microanatomy of skin organs.

## 5. Conclusions

The interactive ST SOM browser provides a new bioinformatics resource to study the spatial architecture of diseased and healthy tissues with gene transcript and cellular resolutions. It enables knowledge mining in the sense of the ‘skopus’, i.e., the ‘look at’ functionality, of microscopic images based on the expression of nearly twenty thousand genes varying across the image and serving as markers for cell types and their varying transcriptional programs. The use cases demonstrate that the detailed look-at option will allow a better understanding of intra-tumoral heterogeneity in solid tumors. The ST patterns of the mouse brain illustrate a use case related to the microanatomy of healthy tissues, which will soon be extended by novel ST data of the sebaceous gland available in the browser. Future addition of spatial transcriptomics samples will eventually enable healthy and diseased tissues, or different samples of the same tumors, to be directly compared.

## Data Availability

oposSOM-Browser can be accessed under www.izbi.uni-leipzig.de/opossom-browser (accessed on 2 May 2024). It currently features 25 datasets, among them 6 referring to spatial transcriptomics. Further datasets will be released together with the accompanying scientific publications. The spatial melanoma and brain data used in this publication can be downloaded from the 10x Genomics resources repository: https://support.10xgenomics.com/spatial-gene-expression/datasets (accessed on 2 May 2024).

## Supplementary Materials

The following supporting information can be downloaded at: https://www.mdpi.com/article/10.3390/cimb46050284/s1, Figure S1: Data workflow of ST: Data was generated using 10x Visium sequencing and followed by preprocessing using spaceranger software; Figure S2: The layout of the spatial browser app consists of three panels; Figure S3: Spot-related information panel when clicking on a spot in the main panel; Figure S4: Gene set expression mapping of immunome and tumor microenvironment (TME) signatures; Figure S5: Gene set expression mapping of epigenetic signatures of gene promoter states of melanocytes; Figure S6: Gene set expression mapping of signatures; Figure S7: Cell cluster specific pathway activity in melanoma (MAPK signaling pathway); Figure S8: Cell cluster specific pathway activity in melanoma (VEGF signaling pathway). References [51,55,63,64,75,76,77,78] are cited in the supplementary materials.
