# Supplementary material for: A Spatial Transcriptomics Browser for Discovering Gene Expression Landscapes across Microscopic Tissue Sections
Source: Curr Issues Mol Biol. 2024 May 13;46(5):4701–20. doi: 10.3390/cimb46050284 (PMC11119626; doi:10.3390/cimb46050284)
Supplement: Supplementary file 1 [file cimb-46-00284-s001.zip › cimb-2956583-supplementary.pdf]

A spatial transcriptomics browser for the interactive discovery of gene and pathway activity patterns, biological functions, and receptor-ligand interactions in tumors and healthy tissues

Maria Schmidt, Susanna Avagyan, Kristin Reiche, Hans Binder, Henry Loeffler-Wirth

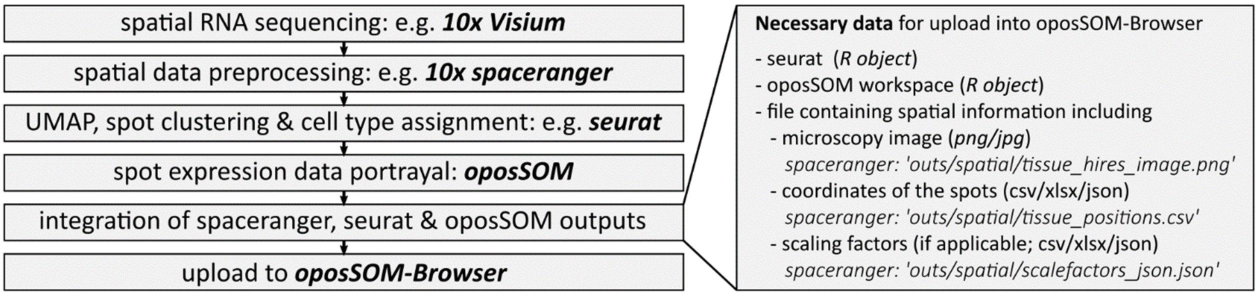

Figure S1. Data workflow of ST: Data was generated using 10x Visium sequencing and followed by preprocessing using spaceranger software. Seurat R-package was then utilized to perform UMAP projection, spot clustering and mapping of cellular identities. This data was input into oposSOM R-package for ST data portrayal. Finally, essential outputs of spaceranger, seurat and oposSOM are compiled one data file and uploaded to oposSOM-Browser: The righthand box lists all data that need to be supplied when interested researchers intent to publish their data in oposSOM-Browser. It comprises the seurat and oposSOM workspaces, and the spatial image together with the coordinates of the spots therein (corresponding spaceranger files are given in italic style).

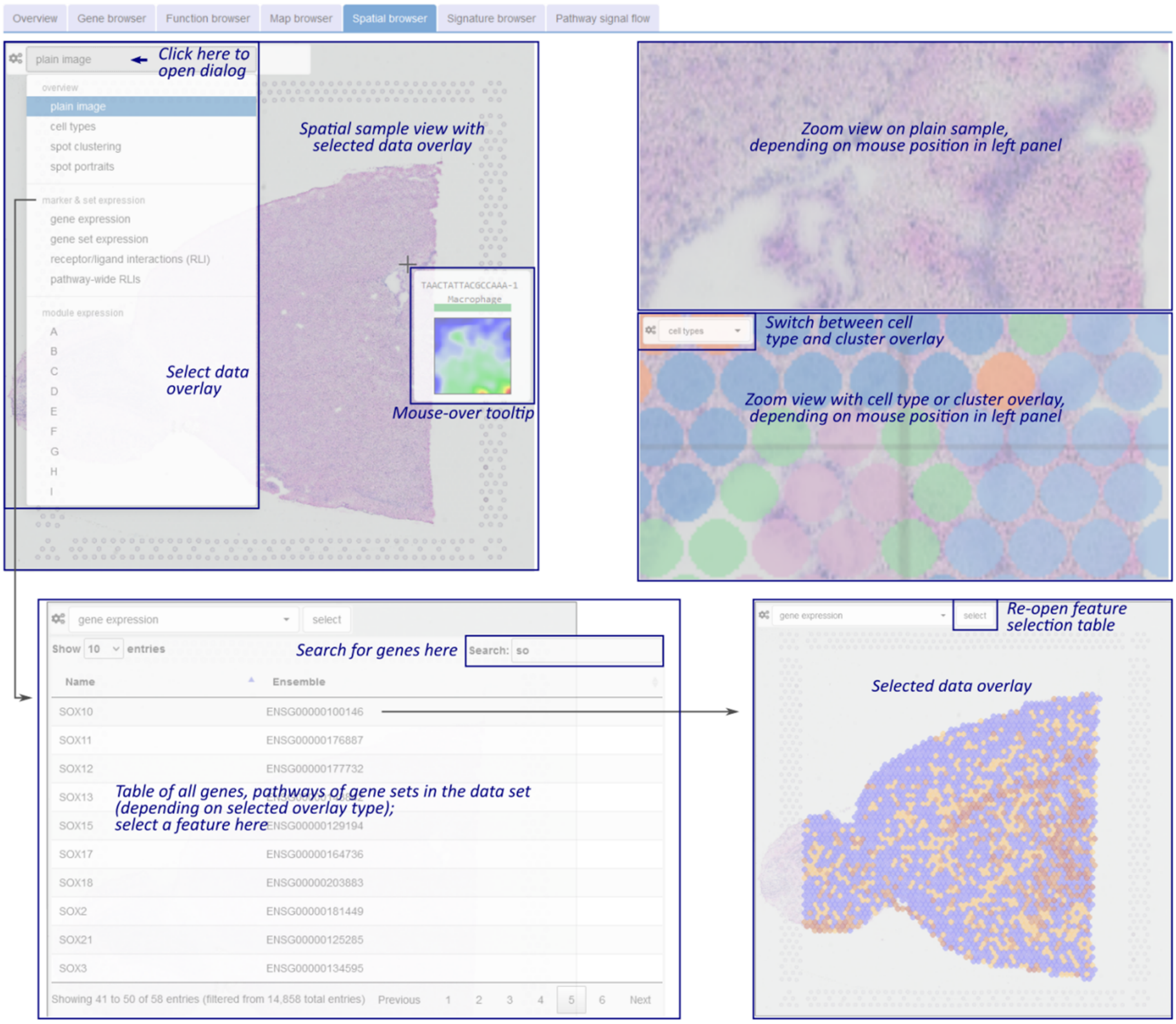

Figure S2. The layout of the spatial browser app consists of three panels: The microscopic image (usually H&E-stained) is shown on the left side, together with the selected data overlay. Enlarged 'zoom' images appear on the right side upon hovering the mouse arrow over this image. Overlay of cell types or spot clusters can be selected (bottom panel). Hoovering the main image also shows the spot barcode, cell type or cluster, and the expression portrait. Several data overlays can be selected: gene, gene set, receptor-ligand, pathway expression, and a selection table.

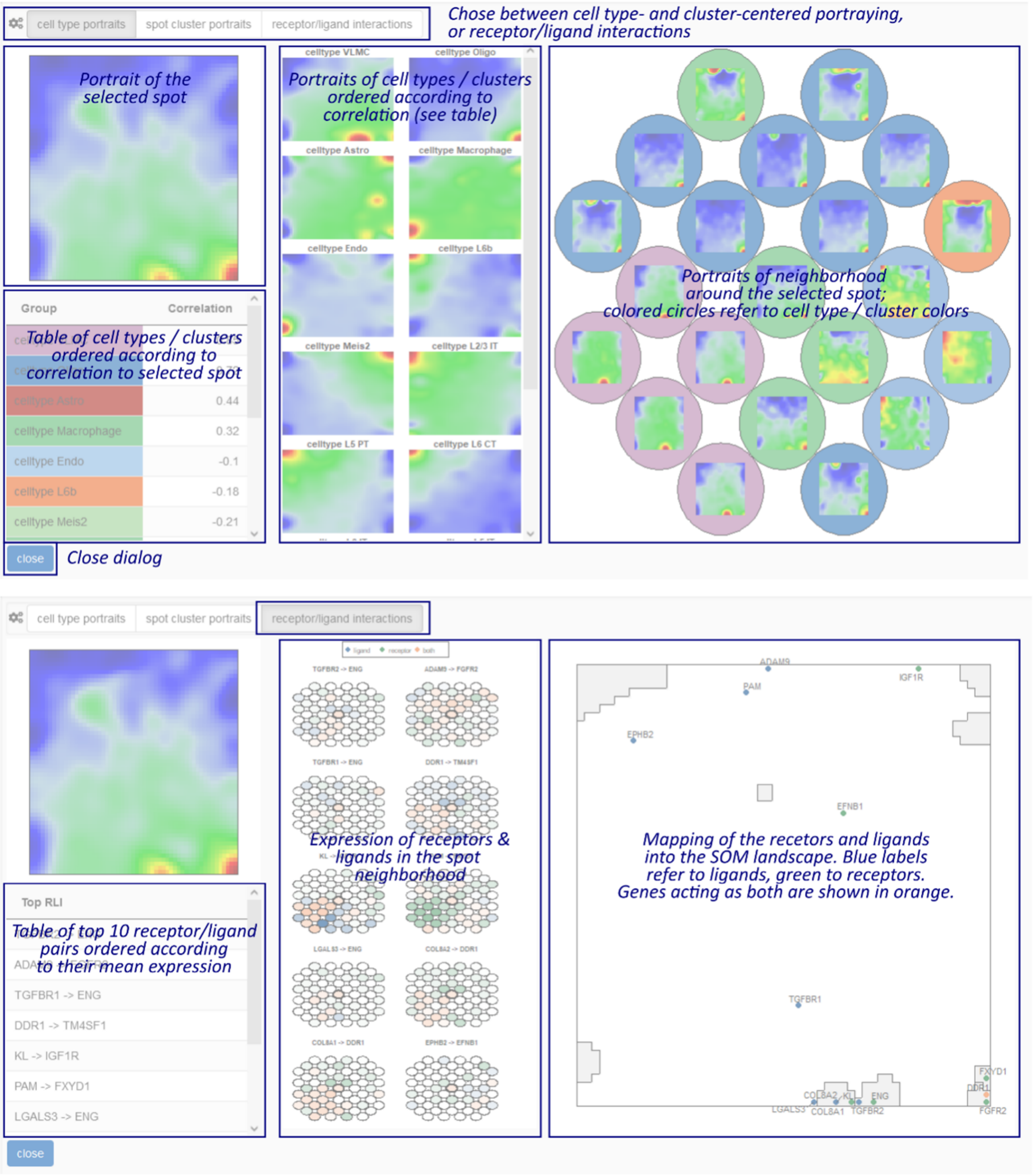

Figure S3. Spot-related information panel when clicking on a spot in the main panel: cell type and cluster portraits, the portraits of the neighboring spots, receptor/ligand expressions of the neighborhood, and their mapping into the SOM landscape.

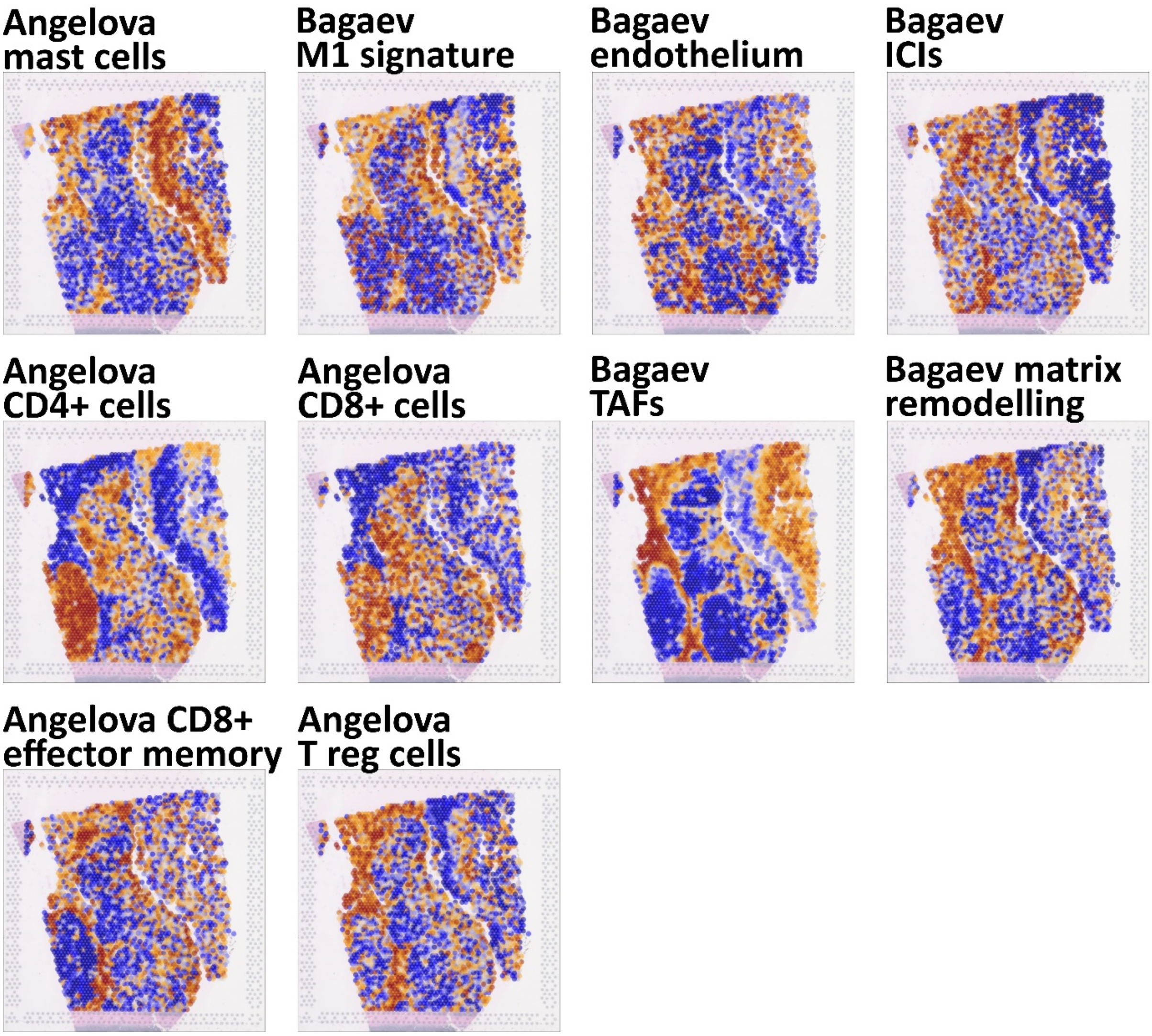

Figure S4. Gene set expression mapping of immunome and tumor microenvironment (TME) signatures taken from the literature [75,76]. Brown areas indicate activation of different immune and stromal signatures in different parts of the image thus illustrating the intratumoral heterogeneity of the TME. Mast cells associate with keratinocyte-rich regions while macrophages type 1 (M1), endothelial signatures, tumor-associated fibroblasts (TAF), effector memory, and Tregulatory cells are found in stroma-rich regions together with a signature assigned to remodeling of the extracellular matrix. CD4+ and CD8+ T cells accumulate in melanocyte/melanoma cell-rich regions however with subtle variations, e.g. CD4+ are found together with highly proliferative melanoma cells.

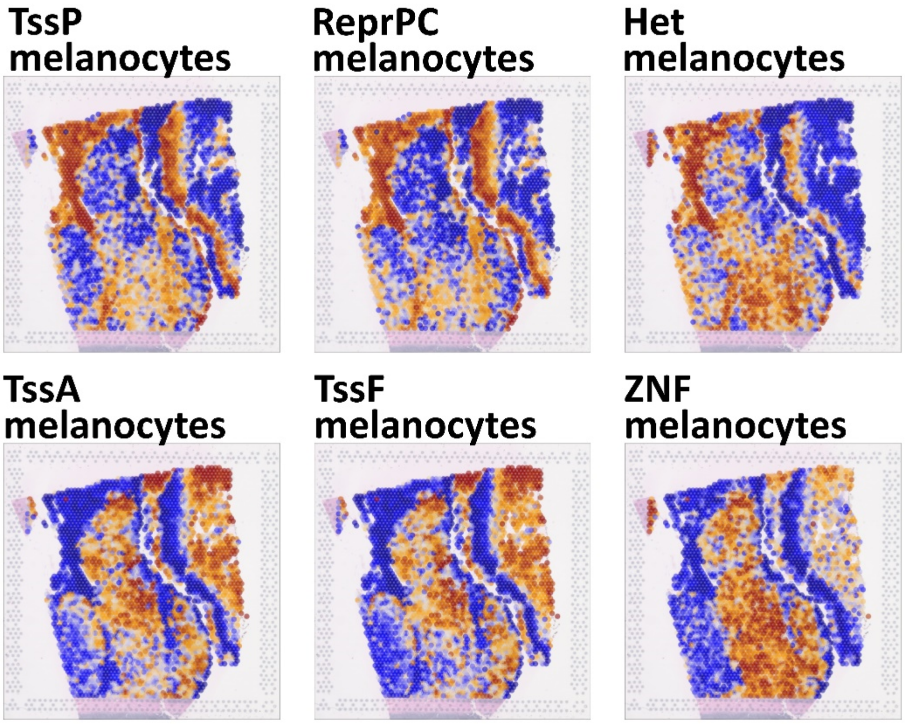

Figure S5. Gene set expression mapping of epigenetic signatures of gene promoter states of melanocytes taken from [77]. Poised (TssP) and repressive (ReprPC) promoter states and partly also heterochromatin (Het) are indicative for stromal-rich areas. Active promoters (TssA) and flanking promoter states (TssF) are found in melanocyte/melanoma cell-rich regions while chromatin accessible to Zink-Finger-Proteins (ZNF) refer also to these areas, however with variable distribution. Note that ReprPC are indicative for H3K27 methylated histones in the promoter region repressing the activity of the downstream gene and TssP refers to bivalent H3K27 and H3K4 methylated histones with partly repressed but "ready-to-go" genes. Both, ReprPC and TssP associate with plastic cell states enabling fate decisions and reprogramming of cell states. In contrast, TssA, TssF, and ZNF refer more to active transcription, e.g. with H3K4 methylated histones in the promoter regions, in more differentiated cell states such as melanoma cells with melanocytic identities.

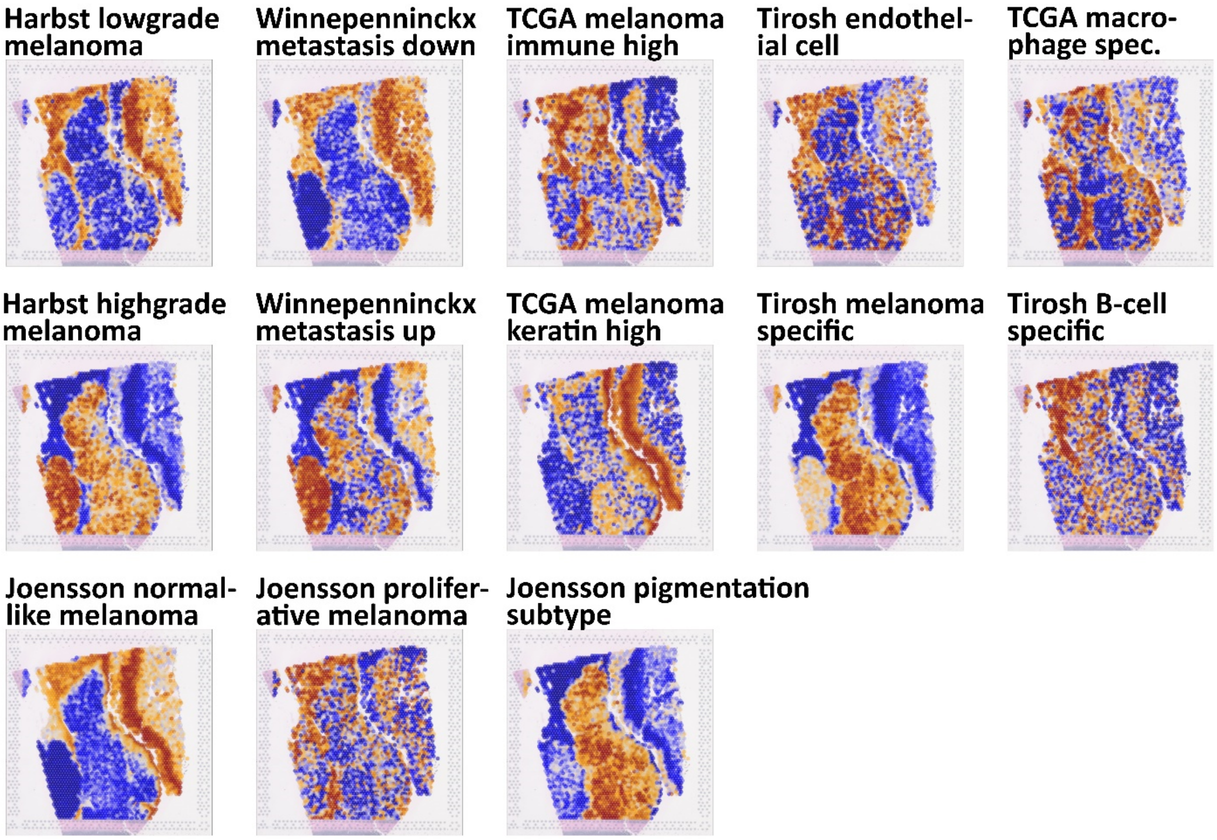

Figure S6. Gene set expression mapping of signatures taken from previous melanoma studies [55,63,64,78]. Signatures of low-grade melanomas upregulate in keratin-enriched areas suggesting a high content of keratinocytes in those tumors. In contrast, highgrade and "pigmentation subtype" melanomas color in brown the central melanocytic part of the image referring to the MITF-program. Metastasis-high and highgrade melanomas associate also with areas of highly proliferative melanoma cells not activated in lowgrade and "metastasis-down" melanomas.

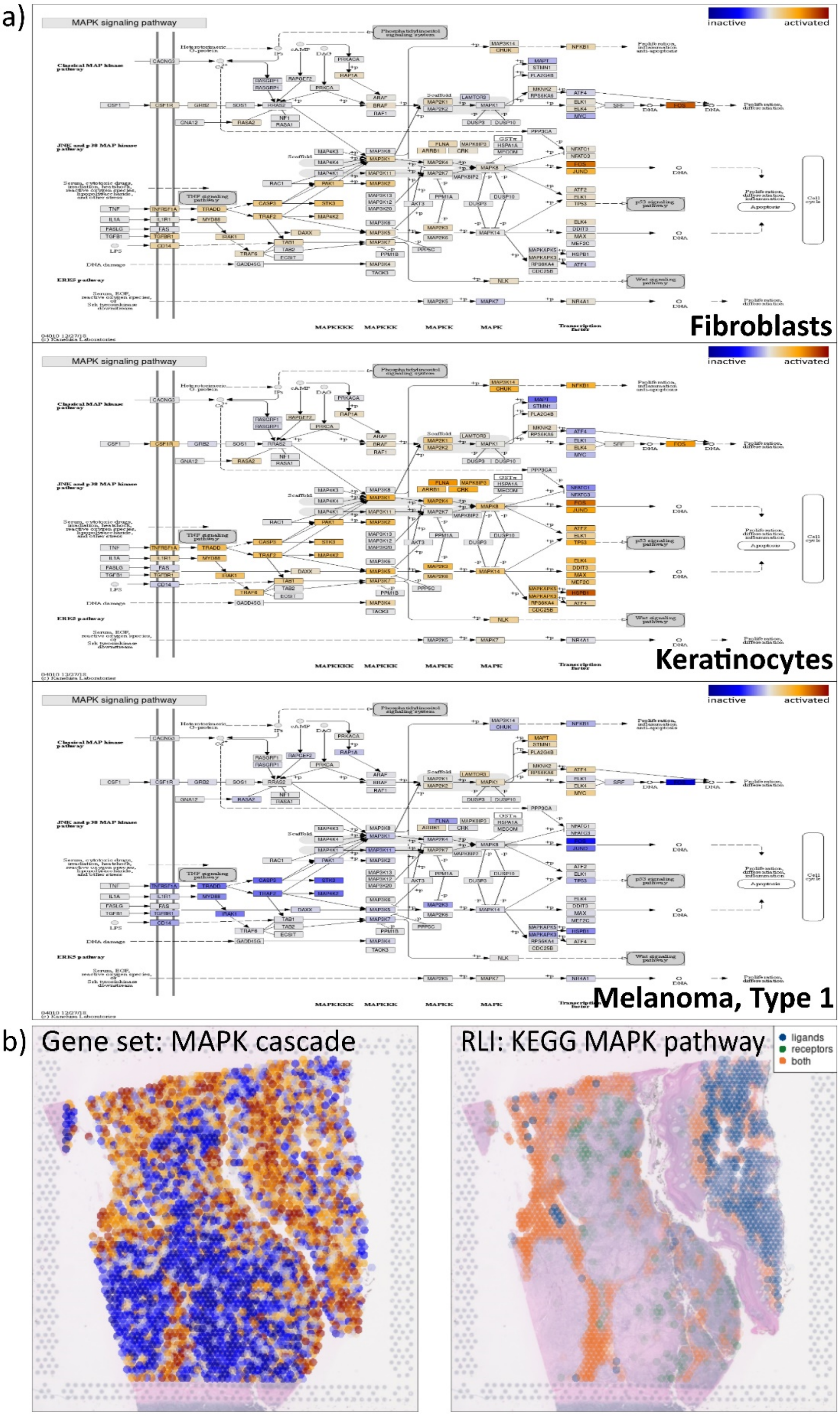

Figure S7. Cell cluster specific pathway activity in melanoma (MAPK signaling pathway): a) The nodes/genes of the KEGG pathway topology are colored in blue to red for inactive to active genes using pathway signal flow metric (PSF, in logarithmic scale) which considers gene expression of upstream genes in the pathway graph [51]. The activation patterns were separately calculated for different cell types. The highest activity is observed in keratinocyte-rich cluster. b) ST image colored according to the activity of the gene set "MAPK activity" and receptor-ligand interactions (RLI) of this pathway. Indeed, keratinocythe-rich clusters c11 and c12 show the highest expression in agreement with the pathway activity in part a. In contrast, joint RLI expression is maximum I type 2 melanoma areas.

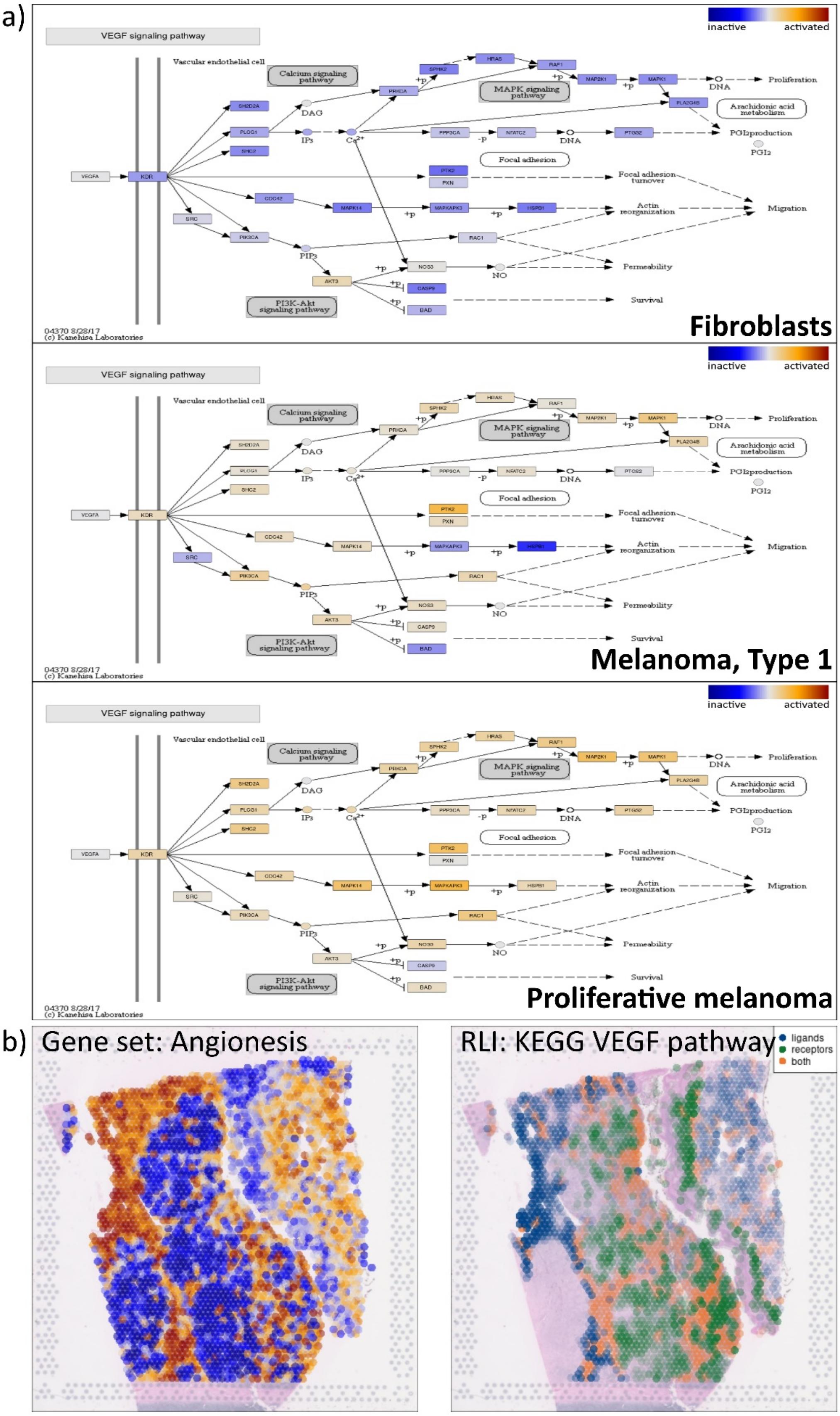

Figure S8. Cell cluster specific pathway activity in melanoma (VEGF signaling pathway): a) PSF-activity in selected cell types/clusters. b) ST image colored according to the activity of the gene set "angiogenesis" and receptor-ligand interactions (RLI) of this pathway. The ST reveals fine structures in the tumor regions.
